# Changes in Diet and Physical Activity among 18–65-Year-Olds after the First National COVID-19 Lockdown in Denmark

**DOI:** 10.3390/nu15061480

**Published:** 2023-03-20

**Authors:** Jeppe Matthiessen, Anja Biltoft-Jensen, Anders Stockmarr, Sisse Fagt, Tue Christensen

**Affiliations:** 1 National Food Institute, Technical University of Denmark, 2800 Kongens Lyngby, Denmark; 2 Department of Applied Mathematics and Computer Science, Technical University of Denmark, 2800 Kongens Lyngby, Denmark

**Keywords:** COVID-19, adults, eating habits, exercise, MVPA, screen time, overweight, Danish

## Abstract

COVID-19 lockdowns affected everyday life significantly and made it challenging to achieve or maintain a healthy lifestyle. The aim of the present study was to examine longitudinal changes in Danish adults’ eating habits and physical activity (PA) assessed during and after the first national lockdown in 2020. Furthermore, changes in body weight were examined during the first lockdown period. The whole diet (semi-quantitative Food Frequency Questionnaire), sociodemographic factors, moderate-to-vigorous intensity PA (MVPA), leisure screen time, anthropometrics, change in body weight, and stress level were assessed with a self-administered web-based questionnaire among 839 18–65-year-old Danes during and 5–6 months after the lockdown. Both favorable (decreased intake of saturated fat) and unfavorable (decreased intake of whole grain and fish, and increased intake of red meat) changes were found in the diet after the lockdown, while favorable changes in MVPA (increase in couples) and leisure screen time (decrease with a combined effect of family status and education) were found for PA. More Danish adults reported to gain weight (mean 3.0 kg) than to lose weight (mean 3.5 kg) during the first lockdown period (27% vs. 15%). The study showed favorable changes in PA and mixed results regarding diet among Danish adults after the lockdown. Furthermore, the first lockdown period unfavorably impacted the body weight of many Danes.

## 1. Introduction

The COVID-19 pandemic is a serious infectious outbreak globally and has so far (7 December 2022) caused 642 million confirmed cases of SARS-CoV-2 and more than 6.6 million deaths [1]. In Europe, 266 million cases and 2.1 million deaths have been confirmed (7 December 2022). As one of the European countries, Denmark implemented several restrictions in the spring of 2020 to prevent spread of the COVID-19. One key restriction was a national lockdown from 11 March to 14 April 2020 that changed everyday life significantly and made it challenging to achieve or maintain a healthy lifestyle. With the lockdown, followed closure of workspaces, day care centers, schools, universities, gyms and sports venues, shopping centers, and restaurants, a ban on social events with more than 10 people, and travel restrictions (Supplementary Materials: Outbreak management of COVID-19). This implied that many Danes had to work from home or were sent home without work, including parental teaching and day-caring for younger children, online education for school children and students, less shopping, social gatherings, and dining out. Despite the restrictions, Danes were allowed to be outdoors and to do grocery shopping during the lockdown period. After the lockdown, restrictions were eased in Denmark to better maintain everyday life with work, education, restaurant visits, leisure time activities, etc. In September–October 2020, up to 50 people were allowed to assemble: restaurants, bars, and cafes were open to 22:00 and conducting smaller social events and private gatherings was allowed. Still, employees were encouraged to work from home and social events in workplaces, schools, leisure-time activities, etc., were cancelled.

Even if restrictions such as national lockdowns, social distancing, and isolation might have been successful to prevent the spread of the COVID-19, these efforts seem to have unintended consequences and may have affected people’s eating habits, physical activity (PA), and body weight unfavorably. It may have also worsened public health and the obesity epidemic in the general population [2,3,4,5]. A Norwegian study reported on more emotional eating of sugar-rich food and drinks among those with COVID-19-related worries and psychological distress [6]. These findings are supported by data from Denmark, where higher intakes of sweet food and drinks were found during the lockdown [7,8,9]. A review showed a decrease in PA level and an increase in sedentary behavior during the COVID-19 lockdowns [3]. A global survey on PA found a 27% reduction in daily step counts after COVID-19 was declared a global pandemic on 11 March 2020 [10].

Several Danish as well as international COVID-19 studies have been published examining changes in selected eating habits and PA during the lockdown compared to before [7,11,12,13,14,15]. A few studies have also assessed the whole diet and PA [16,17,18]. The authors are not familiar with other studies that have assessed changes in the whole diet and PA during and after a national lockdown in the general adult population; thereby, exploring the impact of the lockdown on behavioral changes in diet and PA onwards when restrictions were eased.

The purpose of this study was to examine the effect of the COVID-19 pandemic on Danish adults’ eating habits and PA by collecting data during the first national lockdown (March–April 2020) and again at a follow-up five to six months after, when restrictions were eased (September–October 2020), to compare diet and PA between the two periods in the first year of the pandemic.

Our main aim was to examine:

1. Longitudinal changes in Danish adults’ eating habits and PA assessed during and after the first national lockdown in the same study population.
2. Sociodemographic characteristics and weight status of those with changed eating habits and PA after the lockdown.
3. Changes in body weight during the lockdown period and how these changes were associated with diet, PA, and stress level.

Results from this study can provide new knowledge about how the extent of restrictions during the COVID-19 pandemic have affected eating habits and PA in the general adult population. These data may help to inform public health authorities and be valuable when planning future outbreak management of infectious diseases, so restrictions may go hand in hand with public health prevention. Promotion of the benefits of achieving or maintaining healthy and balanced eating habits and regular PA to strengthen health, wellbeing, and stress management in the general population is crucial in the prevention of infectious diseases such as COVID-19 [19,20,21,22]. Regular moderate-to-vigorous intensity PA (MVPA) strengthens the immune system and is associated with a reduced risk of at least 30% of infectious diseases and mortality and increases the potency of vaccination [23]. Studies from the US and Korea showed that meeting PA guidelines reduces the risk for severe COVID-19 outcomes such as hospitalization, illness, and death significantly [24,25]. Maintaining an optimal nutritional status was also documented as important for the health of the immune system. Intake levels of micronutrients such as vitamin D, C, B12, and iron are inversely associated with higher COVID-19 incidence and mortality [26].

## 2. Materials and Methods

### 2.1. Study Design and Population

The study was conducted longitudinally and included assessment of the whole diet and questions about sociodemographics, PA, screen time, anthropometrics, and stress level during and after the first national lockdown in Denmark. Furthermore, changes in body weight were assessed during the first lockdown period (March to May 2020).

**Figure 1:**
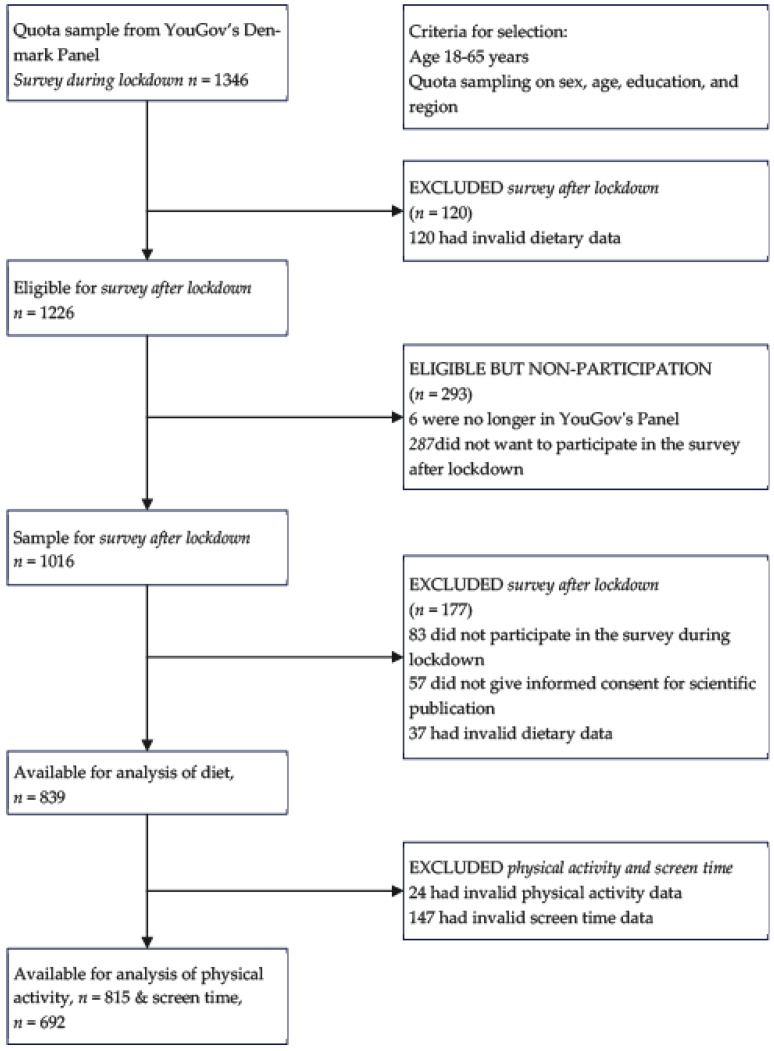
Participant flow chart of the current study.

Two online panel surveys were conducted by the consumer agency YouGov during and after the first COVID-19 lockdown in 2020. The first survey was conducted in the spring of 2020 (3–8 April) and the second in the fall of 2020 (16 September to 1 October). We chose to reassess participants in September 2020 because restrictions were eased in Denmark, and at the time, it was uncertain if previous or new restrictions would be reinstated. Moreover, we were concerned about the loss of web-panelists, making it difficult to reassess diet and PA in the same participants a long time after the lockdown. Other studies showed a significant loss of the initial sample by repeated data collection during the COVID-19 pandemic [27,28]. A self-administered web-based questionnaire was used to collect data in the two surveys. Participants were recruited from YouGov’s Denmark Panel with quota sampling for sex, age, education, and region. YouGov’s Denmark Panel comprises approximately 35,000 Danes aged 15–75 years living in Denmark. Web-panelists aged 18–65 years received an invitation and a link to participate in the study by email. All participants who responded to the first survey during the lockdown were reinvited to the second survey after the lockdown. Participants received an incentive bonus to be exchanged for a gift card, coupon, or participation in raffles. The number of bonus points participants receive depends on the response time of the survey. The average response time to complete the questionnaire was approximately 21 min. in both surveys. Figure 1 shows a participant flowchart of the current study. The population sample contains 839 participants with valid dietary data and 815 participants with valid PA data for the two surveys during and after the lockdown.

### 2.2. Diet

A web-based semi-quantitative food frequency questionnaire (FFQ) was used to assess the whole diet. The FFQ was an updated and modified version of the questionnaire that has been used in the Danish pregnancy planning study. The FFQ from the Danish pregnancy planning study has been validated among Danish females [29]. The validation study shows that the FFQ is appropriate for collecting data on dietary intake, although measures of energy intake is 10% lower compared to the 7-d pre-coded food diary used in The Danish National Survey of Diet and Physical Activity 2011–2013 (DANSDA).

The recall frame in the FFQ was changed from the usual month in the previous year to the last two weeks to capture eating habits during the first national lockdown in 2020. Furthermore, photographs with portion sizes were added. The original questionnaire was also updated with data from market research on retail food sales to extend its use for 18–65-year-old Danes. This includes new food items on the intake of vitamin water, plant-based meat-, fish-, and dairy-alternatives, as well as candy, chocolate, cake, and sweet and salty snacks. Examples of commonly consumed food and drinks in a typical Danish meal pattern were also updated under each item.

In the modified and updated FFQ, participants were asked to record how often they consumed food and drinks in the previous two weeks and the portion size (see Questionnaire). Data on consumption frequencies and the portion sizes of 209 food and 36 drinks were collected. Frequency scales differed between foods and drinks due to a larger variation in foods than drinks consumed. Portion sizes were specified in household measures, e.g., glasses, drinks, slices etc., or estimated from photographs. Eight series of color photographs with portion sizes of food and dishes in a typical Danish meal pattern were included. Intakes of energy, nutrients, food, and drinks were calculated for the study population using the software system webGIES 2019 (National Food Institute, Technical University of Denmark, Kongens Lyngby, Denmark), the recipe collection from DANSDA 2021–2023, and the Danish Food Composition Databank Frida ver. 4, 2019 (National Food Institute, Technical University of Denmark, Kongens Lyngby, Denmark). Validation of the diet recording was done by evaluating extreme estimated dietary intakes in the FFQ. Reporting status of energy was defined according to the Goldberg method, which is used to define mis-reporters of energy intake [30]. The Goldberg method appears to be a reasonable technique for categorizing mis-reporters on a FFQ [31]. Basal Metabolic Rate (BMR) was estimated according to equations from the Nordic Nutrition Recommendations 2012 [32].

The key indicator variable was a dietary index score that evaluates the overall diet quality by means of an index based on five food and nutrient guidelines from the Official Danish Dietary Guidelines 2013: saturated fat (<10 E%), added sugars (<10 E%), fruit and vegetables (≥600 g/10 MJ/day), fish (≥350 g/10 MJ/week), and whole grain (≥75 g/10 MJ/day) [33]. A slightly modified version of the diet index has previously been validated [34]. An individual score between 0 and 1 was calculated according to the compliance with each of the five guidelines. The dietary index score was calculated as the sum of the five scores, ranging from 0 to 5: Most far from compliance to compliance with all five dietary guidelines.

### 2.3. Physical Activity

The Nordic Physical Activity Questionnaire (NPAQ) was used to assess PA and leisure screen time [35]. NPAQ has been used to monitor PA and sedentary behavior in large-scale Danish, Nordic, and international population surveys [36,37,38]. NPAQ has been validated against accelerometry in adults and the validation studies find that the questionnaire reflects the objectively measured levels of PA and is sufficiently reliable and valid to monitor PA in the general population [35,39].

NPAQ comprises two questions on leisure PA spent on MVPA and vigorous intensity PA (VPA) during the last 7 days and two questions on average daily leisure time spent sedentary in front of a TV or computer screen during the last 7 days (see Questionnaire). Moderate intensity PA (MPA) was estimated by subtracting VPA from MVPA. Compliance with guidelines on PA was classified as reporting at least 150 min of MPA per week or at least 75 min of VPA per week or an equivalent combination of MPA and VPA throughout the week [40]. Physically inactive was defined as adults who fail to meet the PA guidelines [32]. Guidelines for processing and classifying data from NPAQ have been described elsewhere [41]. Leisure screen time (TV and computer time) was used as an indicator of sedentary behavior. Very high leisure screen time was defined as individuals spending more than 6 h of their daily leisure time in front of a TV or computer screen. We also used a combined measure of MVPA and leisure screen time that was named ‘Sedentary leisure time’ to examine the public health challenge of those with insufficient PA and sedentary behavior. Sedentary leisure time was defined as individuals that have both been physically inactive and have had very high leisure screen time (>6 h/day). Our key variables were MVPA and leisure screen time.

### 2.4. Anthropometrics, Change in Body Weight, and Stress Level

Questions on self-reported height and weight was used to calculate BMI (kg/m^2^) by dividing weight (kg) by the square of height (m). BMI was used to classify individuals according to their weight status using WHO’s cut-off values for overweight and obesity as indicators of excessive body fat accumulation presenting a health risk [42]. Self-reported weight was also used to estimate BMR [32]. Anthropometrics and self-reported change in body weight during the early period of the pandemic (March to May 2020) covering the first lockdown were only part of the second survey questionnaire. Weight gain or weight loss were reported in intervals of kilograms.

Stress level was assessed by a question that has been used in DANSDA 2011–2013; however, the recall frame was modified from last month to last two weeks to capture stress level during (and after) the lockdown.

### 2.5. Statistical Analyses

Diet, PA, and stress level were analyzed to detect changes in diet and PA assessed during and after the first lockdown using descriptive statistics as the first step. Variables were compared with *t*-tests. We used a level of *p* < 0.05 to indicate statistical significance in all analyses.

The second step comprised statistical modeling that was carried out to identify effects on changes in diet, PA, and stress level in more detail between during and after the lockdown. Effects of sociodemographic factors, weight status, and time (assessed during and after the lockdown) were investigated. Each independent variable was modeled with a main effect (changes in diet, PA, and stress level) and an interaction with time. For continuous dependent variables, the dependent variable was transformed based on results from a box-cox analysis to achieve appropriate normality. For data containing zeroes, data were slightly perturbed prior to box-cox transformations and a sensitivity analysis was carried out. Logistic regression models were used to analyze categorical dependent variables. Dual analyses were performed for ordinal variables (three categories) considering the top category or the collapsed two top categories as dependent variable. A random-effect element was used in all models to handle the paired structure of data obtained during and after the lockdown for the same participant, i.e., a mixed model. We corrected for effect of reporting status of energy by including it as an independent categorical variable in all dietary analyses, where the reference group was acceptable reporters, allowing interaction with time. Results were reported for acceptable reporters, thus correcting other effects for decreased or increased reporting levels of energy for under- and over-reporters. Effect of an independent variable on the difference between time categories (during vs. after the lockdown) was reported if the interaction of the independent variable and time was statistically significant, thereby interpreting such an interaction as effect of the change after the lockdown. The direction of the effect of time was shown for levels of subgroups of significant independent variables, which significantly singled themselves out from other subgroups, e.g., the age group 18–34-year-olds significantly singled itself out from the other age groups for the independent variable added sugars. Non-significant levels were assumed to have no association with time. Green arrows were used to indicate favorable changes and red arrows unfavorable changes. Black arrows indicated a neutral change. If no level of subgroup stood significantly out from the rest for the significant independent variables, only the *p*-value was reported. Odds ratios (OR) and confidence intervals were calculated for categorical dependent variables.

Logistic regression models were also used to analyze Danish adults with a large change in overall diet quality, MVPA, and leisure screen time after the lockdown, defined as their model residual in the original analysis being outside ±1 standard deviation. We analyzed whether sociodemographic factors and weight status impacted the probability of having a large positive or, respectively, negative residual difference in separate analyses.

Moreover, logistic regression analyses were carried out to analyze the effect of sociodemographic factors and weight status on the probability distribution of self-reported weight gain, weight maintenance, or weight loss during the lockdown period. Numerical body weight change was reported with descriptive statistics, and differences in diet, PA, and stress level were analyzed according to self-reported body weight change (gain, maintenance, loss) with independent sample *t*-tests.

Finally, we performed additional analyses: firstly, to compare the diet between mis-reporters and acceptable reporters; and secondly, to compare diet and PA with public health data before the lockdown. Comparison of sociodemographic characteristics and diet among acceptable reporters and mis-reporters of energy during the lockdown were analyzed with *t*-tests and chi-square tests. Additional data for diet and PA before the lockdown from DANSDA 2011–2013 and the Nordic Monitoring System 2014 are presented Supll. Furthermore, we examined the seasonal effect of diet and PA in previous research for reason of comparison with data in the present study. Seasonal variation for diet and PA were analyzed with independent sample *t*-tests, one-way ANCOVA with sex as covariate, and chi-square tests using data from DANSDA 2011–2013.

Statistical analyses were carried out using R version 4.02 (R Core Team (2021)) and IBM SPSS Statistics, IBM Corp., New York, NY, USA version 25.

## 3. Results

### 3.1. Characteristics of the Study Population

Sociodemographic characteristics showed that the study population was close to the distribution of sex, age, education, and region in the general Danish adult population (Table 1). Still, 18–34-year-olds were somewhat under-represented and 50–65-year-olds were somewhat over-represented. Self-reported anthropometrics showed the prevalence of being overweight (including obesity) and obesity among 18–65-year-old Danes were 56.6% and 20.8%, respectively.

All reported results are statistically significant unless otherwise stated when comparing changes between the first lockdown and at a follow-up 5–6 months after.

### 3.2. Change in Diet and Physical Activity Assessed during and after the Lockdown

#### 3.2.1. Diet

Descriptive statistics showed a large decrease in energy intake (0.6 MJ/day) after the lockdown among Danish adults compared to during the lockdown; however, the proportion of under-reporters of energy intake also increased (Table 2). The proportion of mis-reporters of energy intake was high during and after the lockdown due to a high proportion of under-reporters (45% vs. 51%). We found more mis-reporters among males and adults who were overweight/obesity (Table S1). Overall, mis-reporters registered a healthier diet composition than acceptable reporters during the lockdown. A lower percentage of carbohydrate, including added sugars, and a higher percentage of protein was found among mis-reporters. Furthermore, a higher content (g/10 MJ) of fruit and vegetables, fish, and water as well as a lower content of alcoholic drinks was registered among mis-reporters. A less healthy diet composition among mis-reporters was only seen for red meat.

Analyses of macronutrients showed a decrease in the intake of dietary fiber after the lockdown, but otherwise, no change was seen in the macronutrient distribution. We found both favorable and unfavorable changes in the intake of food groups between the two survey periods. Intake of whole grain (unfavorable), fish (unfavorable), and candy and snacks (favorable) decreased after the lockdown, while the intake of water increased (favorable). No changes were found in the overall diet quality (dietary index score) and the consumption of fruit and vegetables, red meat, and sweetened or alcoholic drinks.

The statistical modeling showed an effect of region and education on the change in energy intake after the lockdown. However, no regional or educational group stood out as significant (Table 3). Overall, we found a decrease in the consumption of saturated fat (favorable) and whole grain and fish (both unfavorable), and an increase in the intake of red meat (unfavorable) after the lockdown. The detailed analyses revealed effects for saturated fat, added sugars, and red meat that were not apparent from the crude descriptive statistics. The decrease in the consumption of whole grain was linked to a lower intake of rye bread among acceptable reporters (during: 68 ± 2 g/day vs. after: 57 ± 2 g/day, *p* = 0.001). The group of 18–34-year-olds stood out from other age groups with favorable changes after the lockdown: Their intake of added sugars decreased, and their intake of protein (neutral change) and water increased. Educational level had a significant impact on the change in dietary fiber intake as those with upper secondary school and medium/long higher education or held a Ph.D. decreased their intake after the lockdown (unfavorable). Sex, family status, household income, and weight status had no effect on dietary changes after the lockdown, according to the statistical modeling. We found no change in the overall diet quality, the consumption of total fat, carbohydrates, fruit and vegetables, and candy and snacks, as well as sweetened and alcoholic drinks after the lockdown. The modeled findings were consistent with the descriptive statistics, except for the decreased intake of candy and snacks that could not be confirmed in the more detailed analysis.

#### 3.2.2. Physical Activity and Stress Level

Descriptive statistics showed favorable changes for key PA variables after the lockdown (Table 2). Time spent in MVPA increased after the lockdown (0.5 h/wk) and leisure screen time decreased (0.5 h/d). These changes were mirrored in a decreased proportion of physically inactive (from 43% to 37%), those with very high leisure screen time (from 43% to 37%), and those with sedentary leisure time (from 19% to 13%), respectively. Moreover, time spent in VPA increased and computer time decreased after the lockdown.

Statistical modeling found that couples increased their time spent in MVPA after the lockdown (favorable; Table 3). Overall, Danish adults were less likely to be physically inactive after the lockdown.

We found a decrease in leisure screen time after the lockdown that was moderated by a combined effect of family status and education: Leisure screen time decreased among adults with basic school in both singles and couples and among adults with medium/long higher education or Ph.D. in singles (favorable). Moreover, 35–49-year-olds were less likely to have very high leisure screen time (>6 h/day) after the lockdown (favorable), compared to 18–34- and 50–65-year-olds, who did not show a decrease in very high leisure screen time. Couples without children were more likely to have had very high leisure screen time after the lockdown compared to couples with children and singles (unfavorable). When analyzing sedentary leisure time, i.e., those that have both been physically inactive and have had very high leisure screen time, adults who had normal weight and were overweight were less likely to have had sedentary leisure time after the lockdown (favorable) compared to adults with underweight and obesity who did not show a decrease in sedentary leisure time. Sex, region, and household income had no effect on changes in PA and leisure screen time after the lockdown. Descriptive and modeled findings for key PA variables were overall consistent.

We found no change in stress level after the lockdown. A proportion of 16–17% of Danish adults reported feeling stressed often or all the time during and after the lockdown.

### 3.3. Characteristics of Those with a Large Change in Diet and PA after the Lockdown

Individuals with a large increase or decrease in the overall diet quality (dietary index score), MVPA, and leisure screen time after the lockdown were defined as having a large change in diet or PA: at least +1 SD residual difference corresponds to the 16% of the study population with a large increase, while −1 SD residual difference corresponds to the 16% of the study population with a large decrease.

Sex, family status, and household income had significance for those with a large change in the overall diet quality. Females and individuals with a household income below 600,000 DKK (approximately €81,000) were more likely to have had a large increase in the overall diet quality after the lockdown, compared to men and individuals with a household income of least 600,000 DKK, respectively (favorable; Table 4 upper part). We also found a favorable change in the overall diet quality after the lockdown among couples without children, as they were less likely to have had a large decrease compared to couples with children and singles.

Effects of family status and weight status were found for a large change in MVPA: Adults with obesity were more likely to have had a large increase in MVPA after the lockdown than those who were underweight, normal weight, and overweight (favorable), while no group stood out as significant with a large increase in MVPA regarding family status. However, we found a favorable change in MVPA among couples with children after the lockdown as they were less likely to have had a large decrease in MVPA compared to couples without children and singles.

The large change in leisure screen time were moderated by family status and education: couples without children were both more likely to have had a large increase in leisure screen time and less likely to have had a large decrease after the lockdown compared to couples with children and singles (both unfavorable changes). In contrast, singles with children were more likely to have had a large decrease in leisure screen time after the lockdown compared to singles without children and couples (favorable change). A favorable change in leisure screen time was also found among those with basic school and medium/long higher education or Ph.D. as they were more likely to have had a large decrease compared to those with upper secondary school, vocational education, and short higher education.

Age and region had no effect on a large change in key variables for diet and PA.

### 3.4. Change in Body Weight during the First Lockdown Period and Associations with Diet, Physical Activity, and Stress Level

**Figure 2:**
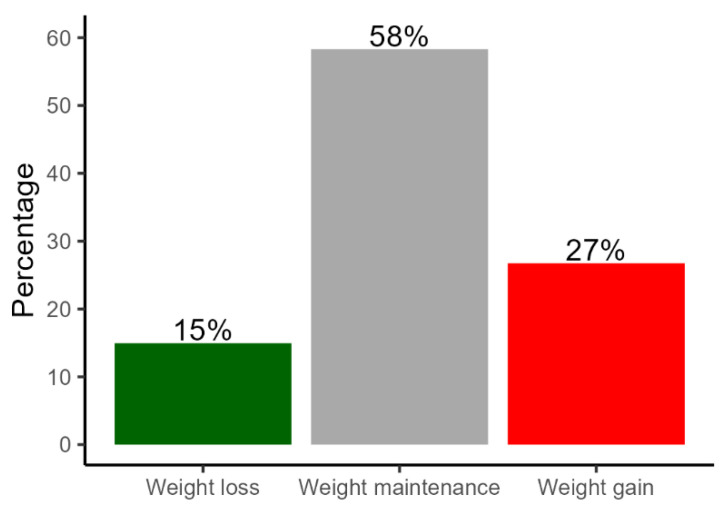
Change in body weight during the first lockdown period among Danish adults (*n* = 789).

A proportion of 27% of Danish adults reported they gained weight during the first lockdown period, while 58% maintained their body weight and 15% reported that they lost weight (Figure 2). On average, weight gainers gained 3 kg and weight losers lost 3.5 kg from March to May 2020 (Table 5). A mean weight gain of 0.3 kg was found among Danish adults during the lockdown period. Results showed that the overall diet quality (dietary index score) and MVPA were lower and leisure screen time higher among weight gainers than among weight maintainers. Moreover, stress level was higher among weight gainers and weight losers compared to weight maintainers. Logistic regression analysis showed that the probability of gaining weight was dependent on sex, age, family status, and weight status (Table 4 lower part). The age range of 50–65-year-olds were less likely to have gained weight than 18–34- and 35–49-year-olds during the lockdown period. Females were more likely than males to have gained weight. Singles without children and adults with overweight or obesity were also more likely to have gained weight compared to singles with children and couples and those with underweight and normal weight, respectively. Overweight adults were also more likely to have lost weight during the lockdown period compared to those who were underweight, normal weight, and obese.

We found no effect of region, education, and household income on change in body weight during the first lockdown period.

## 4. Discussion

The findings of the present study indicate that, five to six months after the first national lockdown in 2020, there were favorable changes in PA among Danish adults, but mixed results regarding their diet. However, the first lockdown period had an adverse effect on the body weight of many Danes. To the best of our knowledge, this is one of the first studies to report on the whole diet, PA, and sedentary behavior during and after a national lockdown with eased restrictions in the general adult population. Consequently, we will primarily compare our results with studies that have investigated changes in diet and PA before and during the COVID-19 lockdown.

After the lockdown, there were both favorable (decreased intake of saturated fat and added sugars (18–34-year-olds) and increased intake of water (18–34-year-olds)) and unfavorable changes in the diet (decreased intake of dietary fiber (upper secondary school and medium/long higher education or Ph.D.), whole grain and fish, and increased intake of red meat). Since 43% of Danish adults worked from home during the first national lockdown, this influenced their eating habits as they spent more time cooking and preparing home-cooked meals [7,8,43]. Open whole grain rye bread sandwiches were frequently eaten with fish as cold cuts for lunch and dinner as some of the home-prepared meals during the lockdown. After the lockdown, more Danes worked at the workplace, which may explain the decrease in the intake of whole grain rye bread and fish. Rye bread is the main contributor to whole grain and fish eaten as cold cuts contributes to half of the total fish consumption among Danish adults [44]. The increase in intake of red meat after the lockdown might be associated with eating out more. Other Danish COVID-19 studies reported a higher degree of emotional eating of sugar-rich foods such as cake during the lockdown period [7,8,9,43]. Our data do not show changes in the consumption of candy and snacks (including cake) and stress level after the lockdown using statistical modeling. However, Danes were world champions in buying candy before the lockdown [45]. This habit is strongly linked to “hygge”—an important aspect of Danish culture and associated with enjoying simple pleasures such as eating candy and watching television and spending time with loved ones such as family members. “Hygge” may have increased during the lockdown but may have been difficult to change after the lockdown. The reported intakes of candy and snacks during and after the lockdown were high. Still, we found a decreased intake of added sugars among 18–34-year-olds after the lockdown. Changes in eating habits after the lockdown were among other factors moderated by age. The group of 18–34-year-olds stood out as a group with favorable changes in the diet. These results may indicate that 18–34-year-olds resumed to healthier eating habits more rapidly after the lockdown than 35–49- and 50–65-year-olds. A large increase in the overall diet quality after the lockdown was found among females and adults with household incomes below 600.000 DKK. The healthier eating habits among females after the lockdown could be explained by inclines in weight loss promoting behaviors as they were more likely to have gained weight during the first lockdown period than men.

When we compare 18–65-year-old Danes’ diet during the COVID-19 pandemic with national data before the pandemic (Table S2 upper part), mixed findings were identified. Eating habits during the pandemic were more favorable compared to the average Danish diet before COVID-19 due to a lower intake of saturated fat, red meat, and alcoholic drinks, but less favorable due to a lower intake of fruit and vegetables and fish, and a higher intake of candy and snacks and sweetened drinks, respectively. This comparison should be viewed in the light of differences between study populations, dietary assessment methods, energy intake, and survey years. Overall, our findings are in line with a systematic review of the global changes in eating habits during the lockdown, except for the lower intake of fruits and vegetables that was also found in other Danish COVID-19 studies [7,8,46].

Favorable changes were found in PA (increased MVPA in couples) and sedentary behavior (decreased leisure screen time with a combined effect of family status and education) among Danish adults after the lockdown. These changes were mirrored in a decreased proportion of physically inactive, those with very high leisure screen time (35–49-year-olds), and those with sedentary leisure time (adults who had normal weight and were overweight), i.e., those that have both been physically inactive and have had very high leisure screen time. A study from Scotland also found decreased sitting time among 18 years or older 2–3 months after the first national lockdown when restrictions started to ease, but no change in MVPA [27]. It is worrying, from a public health point of view, that 460,000–700,000 Danish adults have had sedentary leisure time during and after the first lockdown. On average, Danes spent around 6 h daily of their leisure time in front of a TV or computer screen during the first wave of the COVID-19 pandemic: this value is somewhat above the threshold of 3–4 h/day for increasing the risk of disease and mortality [47]. A large French study found the presence of children at home to be associated with unfavorable changes in PA during the lockdown [17]. Family status also seems to have been a key sociodemographic factor for the changes in PA in our study. We found favorable changes in MVPA after the lockdown among couples and in leisure screen time among couples with basic school, but also among singles with basic school or medium/long higher education and a Ph.D. The favorable change in MVPA may suggest that couples increased their level of exercise and sports activities more rapidly than singles after the lockdown. Our findings also indicate that favorable changes in PA and sedentary behavior do not always go hand in hand for all population groups, as couples without children have had an increase in MVPA and a large increase in leisure screen time after the lockdown.

A comparison of PA and sedentary screen time in the Danish adult population during the pandemic with national data before shows that the proportion of physically inactive (before: 26–34% vs. during: 37–55%) and those with very high leisure screen time (>6 h/day: before: 9–16% vs. during: 37–43%) were higher during the pandemic [36,48] (Table S2 lower part). Danish adults have, on average, spent 0–1.4 h less weekly on MVPA and 1.7–3.0 h more daily on sedentary leisure screen time during the pandemic. These data suggest that many Danes have used the increased ‘free’ time during the pandemic by being sedentary in front of a TV or computer screen. Overall, results indicate that the COVID-19 pandemic has had a highly unfavorable effect on PA and sedentary behavior in the Danish adult population. Our findings are in line with previous research [3,4,10,17,36,38,49,50]. Less exercise and sports activities, as well as less active transportation, especially among the large number of Danes working from home, may explain the decrease in MVPA during the pandemic. A French study showed that active transport such as walking and bicycling was among the activities that was most affected by restrictions during the lockdown [51]. Even 2 years after the beginning of COVID-19, worldwide and European daily step counts have not returned to pre-pandemic levels, indicating that unfavorable changes of PA during the pandemic have become established habits for some time [52]. Of public health concern is that adults may never return to pre-pandemic PA levels.

It is also a public health concern that approximately one in five Danish adults live with obesity due to the adverse health effects that, among other factors, also comprise a higher risk for severe COVID-19 related disease and mortality [19,53]. The prevalence level of overweightness, including obesity, in the present study is comparable to data from the Danish National Health Profile 2021 [36]. We found more Danish adults that reported gaining weight than losing weight during the first lockdown period (27% vs. 15%). On average, weight gainers put on 3.0 kg and weight losers lost 3.5 kg. Our findings are consistent with the documented global trend of weight gain during the first COVID-19 lockdown period [50,54]. Still, results from 4.25 million US adults showed that mean weight gain in first year of the COVID-19 pandemic was small (0.1 kg) [55]. In our study, an average weight gain of 0.3 kg was found among Danish adults in the first three months of the pandemic. According to previous research, one explanation for the high proportion of weight gainers could be that social isolation during the lockdown period might have deteriorated psychosocial health, altered eating behavior in the direction of more snacking, decreased exercise, and increased sedentary time [2,5,50]. Our data confirm that weight gainers had less healthy eating habits, less MVPA and more screen time during the first lockdown period compared to weight maintainers. On average, weight gainers spent 1 h less per week on MVPA and 0.8 h more per day on leisure screen time than weight maintainers. We also found higher levels of stress among weight gainers and weight losers than among weight maintainers during the lockdown period which is in line with other studies [56]. Females, singles without children, and adults with overweight or obesity were more likely to have gained weight during the first lockdown period. These population groups could therefore have been more exposed to COVID-19 related stress that can lead to emotional eating and reduction in PA, thus causing declines in weight gain protective behaviors [6,56,57].

The finding of females to be more likely to have gained weight than men is consistent with other studies that found females to be at increased risk for weight gain due to increases in unhealthy lifestyle behaviors during the lockdown period [2,58]. School and daycare closures, followed by childcare and homeschooling, may have affected females more as primary childcare givers. Singles without children were also more likely to have gained weight during the lockdown period. These data are supported by a French study showing that living alone was a strong risk factor for unhealthy behavior during the lockdown [59]. However, in contrast to what has been reported elsewhere, we found 50–65-year-olds to be less likely to have gained weight during the lockdown period than 18–34- and 35–49-year-olds [2]. We found that adults who were overweight and obese were more likely to have gained weight during the lockdown than those who were underweight and had normal weight. These results align with previous research [2]. Studies from the UK showed that individuals living with obesity were most likely to report declines in weight gain protective behaviors such as eating healthily, exercising, and getting sufficient sleep during the first COVID-19 lockdown, which may have increased their risk of weight gain [12,60,61]. However, we also found favorable changes in PA after the lockdown among adults living with obesity that were more likely to have had a large increase in MVPA compared to those who were underweight, had normal weight, and obese. These findings support research from the UK, where adults living with obesity increased their PA and were attempting to lose weight after the first COVID-19 lockdown [62]. Adults who were overweight were both more likely to have gained and lost weight during the lockdown period than those in other categories: underweight, normal weight, and obesity. These data suggest that the lockdown may have resulted in a polarized effect among adults who were overweight with both weight gainers and losers.

## 5. Strengths and Limitations

One of the strengths in the present study is the longitudinal design that makes it possible to examine changes in eating habits and PA assessed during and after the first lockdown. Another strength is the study sample that was close representative of the general Danish adult population regarding sex, age, education, and region. This increases the generalizability of the results. Data collection with validated questionnaires to assess diet and PA over a short period (6–16 days) may also be viewed as a strength when the aim is to get a snapshot of current eating habits and PA during and after the lockdown. The short recall frames might have increased participants’ ability to accurately recall their diet (last two weeks) and PA (last 7 days). A further strength is the assessment of the whole diet that reflects the usual eating habits. Most studies reporting on eating habits during the COVID-19 pandemic have used indicator questions and therefore did not assess the whole diet [7,8,11,12,13,14,15]. It is also a strength that our findings increase the limited knowledge in Denmark regarding sociodemographic factors associated with changes in eating habits and PA after the lockdown and changes in body weight during the first lockdown period.

There are several limitations in this study. One limitation is the large percentage of excluded participants (38–39%) from the initial sample caused by dropout, invalid dietary or PA data, non-participation in both surveys, and for not giving informed consent for scientific publication. The high response time to complete the questionnaire may be one of the reasons for dropping out and for not reporting valid dietary and PA data. A large percentage of excluded participants was also found in other Danish and international COVID-19 online surveys [8]. Despite the large number of excluded participants in the present study, the sample was still close to be representative for the general Danish adult population.

Another limitation is the high proportion of under-reporters of energy intake; however, we accounted for misreporting in the statistical modeling. Correction of misreporting was important in the present study as under-reporters registered a healthier diet than acceptable reporters. Under-reporting is a common problem with FFQ’s [63] because they do not cover all participants’ usual eating habits perfectly. Furthermore, it may have been challenging for participants to report their intake of food and drinks with a frequency scale that was somewhat different for food and drinks and with no personal instruction before fulfilling the first FFQ. Still, we believe that overall, the dietary assessment method reflected Danish adults’ eating habits during the COVID-19 pandemic quite well. Our data also place a question mark about the interpretation of the size of change in energy intake with descriptive statistics when under-reporting is high and increases between survey periods. This may also be the case for the decreasing intake of candy and snacks after the lockdown that we found with descriptive statistics but that could not be confirmed with the statistical modeling.

A limitation with the assessment of PA and sedentary behavior could be self-report bias, leading to over-reporting of MVPA and under-reporting of sedentary screen time [64]. However, screen time may not have been underestimated that much; it cannot be ruled out that even if TV and computer time have been questioned separately, participants may have reported some TV and computer time that took place simultaneously. Self-report bias has likely been present in both surveys, making data comparable during and after the lockdown.

A final but important limitation is to separate a seasonal effect on diet, and especially PA, from the COVID-19 effect. A scoping review documented a seasonal difference in MVPA, but not in sedentary behavior between spring and autumn, because people tend to be more active in spring than autumn [65]. Still, weather reports showed that it was less attractive to be physically active outside in Denmark in March–April 2020, during the lockdown, compared to after the lockdown, in September 2020. This is because it was eight degrees Celsius colder on average, even if it was less rainy and with more hours of sunshine [66]. Additional analyses of the seasonal effect in PA in Denmark showed no difference in MVPA and the proportion of physically inactive, but 0.5 h/day more leisure screen time in March–April compared to September (Table S3 lower part). Comparison of the results from the present study with additional seasonal data suggest that the increase in MVPA after the lockdown may be attributed to a COVID-19 effect with easing restrictions, whereas the decrease in leisure screen time may be a combined COVID-19 and seasonal effect. Changes in diet may be attributed to a COVID-19 effect (Table S3 upper part).

## 6. Conclusions

This study shows that the extent of restrictions during a pandemic, such as COVID-19, has a significant impact on people’s eating habits, PA, and body weight. Therefore, future outbreak management should prioritize promoting healthy eating and regular PA during a pandemic, taking public health concerns such as unhealthy diet, physical inactivity, and overweightness into account. Additionally, we identified sociodemographic factors that were associated with changes in diet, PA, and body weight after the lockdown, which could guide public health authorities in targeting specific sub-populations during pandemic conditions, such as COVID-19.

## Figures and Tables

**Table 1:** Sociodemographic characteristics and weight status of the study population (*n* = 839) and the Danish adult population (18–65 y).

	Study Population % (*n*)	Danish Adult Population %
Sex		
Male	49.3 (*n* = 414)	49.5
Female	50.7 (*n* = 425)	50.5
Age		
18–34 y	29.0 (*n* = 243)	35.6
35–49 y	31.5 (*n* = 264)	30.6
50–65 y	39.6 (*n* = 332)	33.7
Education		
Basic school (<12 y)	7.3 (*n* = 61)	7.9
Upper secondary school (12 y)	13.6 (*n* = 114)	14.6
Vocational education (13 y, practical)	22.2 (*n* = 186)	20.9
Short higher education (13–14 y)	11.9 (*n* = 100)	11.3
Medium/long higher education, Ph.D. (≥15 y)	45.1 (*n* = 378)	45.3
Region		
Capital Region	32.5 (*n* = 273)	32.9
Zealand	14.4 (*n* = 121)	13.8
South Denmark	21.3 (*n* = 179)	20.6
Central Jutland	21.9 (*n* = 184)	22.7
North Jutland	9.8 (*n* = 82)	10.0
Household income		
<300,000 DKK ^#^	19.4 (*n* = 163)	-
300,000–599,999 DKK	28.6 (*n* = 240)	-
600,000–899,999 DKK	22.1 (*n* = 185)	-
≥900,000 DKK	15.3 (*n* = 128)	-
Unknown/Do not want to answer	14.7 (*n* = 123)	-
Family status		
Single without children <16 y	29.1 (*n* = 244)	-
Single with at least one child <16 y	2.9 (*n* = 24)	-
Couple without children <16 y	47.3 (*n* = 397)	-
Couple with at least one child <16 y	20.7 (*n* = 174)	-
Weight status (*n* = 734)		
Underweight (BMI <18.5)	2.0 (*n* = 15)	-
Normal weight (BMI 18.5–24.9)	41.3 (*n* = 303)	-
Overweight (BMI (25.0–29.9)	35.8 (*n* = 263)	-
Obese (BMI ≥30)	20.8 (*n* = 153)	-

^#^ 7.44 DKK = €1 (9 February 2023).

**Table 2:** Change in diet, physical activity, and stress level among Danish adults assessed during and after the lockdown using descriptive statistics (mean/percentage ± SEM).

	During the Lockdown	After the Lockdown	*p*
Diet (*n* = 839)			
Dietary index score ^#^	3.2 ± 0.1	3.3 ± 0.1	0.38
Energy (MJ/d)	8.5 ± 0.2	7.9 ± 0.2	<0.0001 ***
Reporting status of energy ^&^ (*n* = 739)			0.003 **
Under-reporters (%)	40.1 ± 3.5	48.3 ± 3.6	
Acceptable reporters (%)	55.5 ± 3.6	48.8 ± 3.6	
Over-reporters (%)	4.5 ± 1.5	2.8 ± 1.2	
Total fat (E%)	34.9 ± 0.3	34.8 ± 0.4	0.70
Saturated fat (E%)	13.0 ± 0.2	12.8 ± 0.2	0.12
Carbohydrate (E%)	47.1 ± 0.4	46.9 ± 0.4	0.65
Added sugars (E%)	9.6 ± 0.4	9.2 ± 0.4	0.27
Dietary fiber (g/day)	20.0 ± 0.6	18.5 ± 0.5	0.0003 ***
Protein (E%)	16.0 ± 0.2	16.2 ± 0.2	0.11
Fruit and vegetables (g/day)	265 ± 11	256 ± 10	0.22
Whole grain (g/day)	60 ± 3	54 ± 2	0.001 **
Fish (g/day)	19 ± 1	18 ± 1	0.03 *
Red meat (g/day)	77 ± 3	79 ± 4	0.47
Candy and snacks ^”^ (g/day)	94 ± 5	85 ± 4	0.02 *
Water (g/day)	858 ± 39	923 ± 40	0.02 *
Sweetened drinks ^¤^ (g/day)	425 ± 39	402 ± 37	0.40
Alcoholic drinks ^!^ (g/day)	137 ± 18	142 ± 19	0.72
Physical activity (PA) (*n* = 815)			
Moderate-to-vigorous intensity PA (MVPA; h/wk)	3.5 ± 0.3	4.0 ± 0.3	0.03 *
Moderate intensity PA (MPA; h/wk)	2.3 ± 0.2	2.5 ± 0.2	0.21
Vigorous intensity PA (VPA; h/wk)	1.3 ± 0.1	1.5 ± 0.2	0.02 *
Physically inactive ^£^ (%)	42.5 ± 1.7	37.3 ± 1.6	0.03 *
Leisure screen time (h/day) (*n* = 692)	6.3 ± 0.2	5.8 ± 0.3	0.005 **
TV (h/day)	3.7 ± 0.2	3.4 ± 0.2	0.07
Computer (h/day)	2.6 ± 0.2	2.3 ± 0.2	0.03 *
Very high leisure screen time (>6 h/day; %) (*n* = 692)	42.8 ± 1.8	37.3 ± 1.7	0.04 *
Sedentary leisure time ^$^ (%) (*n* = 692)	19.4 ± 1.2	12.9 ± 0.8	0.001 **
Stress level (*n* = 820)			0.25
Not at all or rarely (%)	57.7 ± 3.4	53.7 ± 3.4	
Occasionally (%)	26.2 ± 3.0	29.1 ± 3.1	
Often or all the time (%)	16.1 ± 2.5	17.2 ± 2.6	

Significant values are shown with asterisks: (*p* < 0.10) * (*p* < 0.05), ** (*p* < 0.01), *** (*p* < 0.001). ^#^ The overall diet quality was evaluated by means of a diet index score based on five food and nutrient guidelines from the Official Danish Dietary Guidelines 2013: Saturated fat (<10 E%), added sugars (<10 E%), fruit and vegetables (≥600 g/10 MJ/day), fish (≥350 g/10 MJ/week) and whole grain (≥75 g/10 MJ/day). ^&^ Under-reporters: EI/BMR < 1.10; acceptable reporters: EI/BMR 1.10–2.19; over-reporters: EI/BMR ≥ 2.20. ” Sweets, chocolate, cake, biscuit, snack bar, ice cream, desserts, and snacks. ^¤^ Sugar sweetened and artificially sweetened soft drinks, energy drinks, cordials, and iced tea. ^!^ Beer, wine, and other alcoholic drinks (liqueur, spirits, alcopops). ^£^ Failure to meet the PA guidelines. ^$^ Physically inactive with very high leisure screen time (>6 h/day).

**Table 3:** Change in diet and physical activity among Danish adults moderated by sociodemographic characteristics and weight status assessed during and after the lockdown using statistical modeling.

	Intercept	Age	Region	Family Status	Education	Weight Status
Diet (*n* = 739)						
Energy (MJ/d)			*p* = 0.01 ^†^		*p* = 0.04 ^†^	
Saturated fat (E%)	*p* = 0.01; ↓ *					
Added sugars (E%)		*p* = 0.01; 18–34 y ↓ **				
Dietary fiber (g/day)					*p* = 0.048; Upper secondary school ↓ ***; Medium/long higher education or Ph.D. ↓ *	
Protein (E %)		*p* = 0.006; 18–34 y ↑ **				
Whole grain (g/day)	*p* = 0.04; ↓ *					
Fish (g/day)	*p* = 0.002; ↓ **					
Red Meat (g/day)	*p* = 0.002; ↑ **					
Water (g/d)		*p* = 0.02; 18–34 y ↑ ***				
Physical activity						
MVPA (h/wk) (*n* = 815)				*p* = 0.005; Couple without children < 16 y ↑ **; Couple with children < 16 y ↑ ***		
Physically inactive ^£^ (%) (*n* = 815)	*p* = 0.03; ↓ * (OR = 0.75 (0.57; 0.99))					
Leisure screen time ^#^ (h/day) (*n* = 692)				*p* = 0.049; Basic school: Single without children < 16 y ↓ ***; Single with children < 16 y ↓ ***; Couple without children < 16 y ↓ *; Couple with children < 16 y ↓ * Medium/long higher education or Ph.D.: Single without children < 16 y ↓ ***; Single with children < 16 y ↓ *		
Very high leisure screen time (>6 h/day; %) (*n* = 692)		*p* = 0.048, 35–49 y ↓ *; (OR = 0.49 (0.28; 0.87))		*p* = 0.03; Couple without children < 16 y ↑ ** (OR = 1.86 (1.12; 3.11))		
Sedentary leisure time ^$^ (%) (*n =* 692)						*p* = 0.04; Normal weight ↓ ***; (OR = 0.33 (0.18; 0.61)); Overweight ↓ **; (OR = 0.31 (0.21; 0.73))

Significant values are shown with asterisks: * (*p* < 0.05), ** (*p* < 0.01), *** (*p* < 0.001). Only factors on diet, physical activity, and sociodemographics with significant effect are shown. *p*-values denote the effect of the intercept or variable. Significant values (asterisks) for subgroups are indicated in relation to non-significant groups. Green arrows indicate a favorable change and red arrows an unfavorable change. Black arrows indicate a neutral change. ORs’ are shown in relation to what the arrows indicate. ^†^ No group stood out as significant. ^£^ Failure to meet the PA guidelines. ^#^ The combined effect of family status and education regarding the decrease in leisure screen time after the lockdown are shown in the columns for both ‘Family status’ and ‘Education’. ^$^ Physically inactive with very high leisure screen time (>6 h/day).

**Table 4:** Sociodemographic characteristics and weight status in Danish adults with a large change in the overall diet quality, MVPA, and leisure screen time after the lockdown using statistical modeling. Furthermore, sociodemographic characteristics of Danish adults with change in body weight during the lockdown period.

	Sex	Age	Family Status	Education	Household Income	Weight Status
Dietary index score ^#^ (*n* = 734)	*p* = 0.02 (+1 SD diff. = increase); Females ↑ * (OR = 1.79 (1.20; 2.69))		*p* = 0.002 (−1 SD diff. = decrease); Couple without children < 16 y ↑ ** (OR = 0.48 (0.30; 0.75))		*p* = 0.01 (+1 SD diff. = increase); <300,000 DKK ^##^ ↑ ** (*OR* = 2.17 (1.26;3.76)) 300.000–599.999 DKK ↑ ** (OR = 2.03 (1.24;3.35))	
MVPA (h/wk) (*n* = 723)			*p* = 0.02 (−1 SD diff. = decrease); Couple with children < 16 y ↑ *; (OR = 0.59 (0.38; 0.92)) *p* = 0.01 ^†^ (+1 SD diff. = increase)			*p* = 0.01 (+1 SD diff. = increase); Obese ↑ ** (OR = 1.77 (1.22; 2.57))
Leisure screen time ^$^ (h/day) (*n* = 578)			*p* = 0.0003 (−1 SD diff. = decrease); Single with children < 16 y ↓ ** (OR = 4.42 (1.61; 12.11)) Couple without children < 16 y ↑ ** (OR = 0.62 (0.42; 0.94)) *p* = 0.02 (+1 SD diff. = increase); Couple without children < 16 y ↑ * (OR = 2.00 (1.17; 3.43))	*p* = 0.0002 (−1 SD diff. = decrease); Basic school ↓ *** (OR = 4.70 (2.32; 9.53)) Medium/long higher education or Ph.D. ↓ ** (OR = 1.90 (1.24; 2.93))		
Weight gain (*n* = 671)	*p* = 0.005; Females ↑ ** (OR = 1.69 (1.18; 2.50))	*p* < 0.0001; 50–65 y ↓ *** (OR = 0.40 (0.27; 0.61))	*p* = 0.04; Single without children < 16 y ↑ ** (OR = 1.79 (1.20; 2.67))			*p* < 0.0001; Overweight ↑ *** (OR = 3.19 (2.05; 4.97)) Obese ↑ *** (OR = 2.77 (1.70; 4.53))
Weight loss (*n* = 578)						*p* = 0.01; Overweight ↑ * (OR = 1.72 (1.06; 2.81))

Significant values are shown with asterisks: * (*p* < 0.05), ** (*p* < 0.01), *** (*p* < 0.001). Only sociodemographic factors with significant effect are shown. *p*-values denotes the effect of the variable. Significant values (asterisks) for subgroups are indicated in relation to non-significant groups. Green arrows indicate a favorable change and red arrows an unfavorable change. ORs’ are shown in relation to what the arrows indicate. ^#^ The overall diet quality was evaluated by means of a diet index score based on five food and nutrient guidelines from the Official Danish Dietary Guidelines 2013: Saturated fat (<10 E%), added sugars (<10 E%), fruit and vegetables (600 g/10 MJ/day), fish (≥350 g/10 MJ/week) and whole grain (≥75 g/10 MJ/day). ^$^ TV and computer leisure time. ^##^ 7.44 DKK = €1 (9 February 2023). ^†^ No group stood out as significant.

**Table 5:** Weight change among Danish adults during the lockdown period and differences in diet, physical activity, and stress level according to change in body weight using descriptive statistics (mean/percentage ± SEM).

	Weight Loss	Weight Maintenance	Weight Gain
Weight change (kg) (*n* =317)	3.5 ± 0.2 (*n* = 106)	-	3.0 ± 0.1 (*n* = 211)
Diet			
Dietary index score ^#^ (*n* = 789)	3.3 ± 0.1 ^a,b^	3.3 ± 0.0 ^b^	3.1 ± 0.1 ^a^
Physical activity			
MVPA (h/wk) (*n* = 778)	4.1 ± 0.4 ^a,b^	4.4 ± 0.2 ^b^	3.4 ± 0.3 ^a^
Leisure screen time ^$^ (h/day) (*n* = 706)	5.8 ± 0.4 ^a,b^	5.7 ± 0.2 ^b^	6.5 ± 0.3 ^a^
Stress level			
Often or all the time (%) (*n* = 781)	25 ± 4 ^b^	13 ± 2 ^a^	22 ± 3 ^b^

^a,b^ Estimates with unlike superscript letters differed significantly (*p* < 0.05) according to change in body weight. ^#^ The overall diet quality was evaluated by means of a diet index score based on five food and nutrient guidelines from the Official Danish Dietary Guidelines 2013: Saturated fat (<10 E%), added sugars (<10 E%), fruit and vegetables (≥600 g/10 MJ/day), fish (≥350 g/10 MJ/week) and whole grain (≥75 g/10 MJ/day). ^$^ TV and computer leisure time.

## Data Availability

In accordance with Danish law and GDPR, the data and the analytical scripts used in the present study can only be accessed through the servers at The Technical University of Denmark and requires a Disclosure Declaration. Access and a Disclosure Declaration can be granted upon request if the applicant fulfils the criteria for access. The Technical University of Denmark can be contacted by email: jmat@food.dtu.dk.

## Supplementary Materials

The following are available online at https://www.mdpi.com/article/10.3390/nu15061480/s1, Questionnaire; Outbreak management of COVID-19; Table S1: Sociodemographic characteristics, weight status and diet among acceptable reporters and mis-reporters (under- and over-reporters) of energy during the lockdown; Table S2: Diet and physical activity among 18–65-year-olds in the Danish National Survey of Diet and Physical Activity 2011–2013 (DANSDA 2011–2013) and the Nordic Monitoring System 2014 (NORMO 2014); Table S3: Seasonal variation between March-April and September for diet and physical activity among 18–65-year-old Danes in the Danish National Survey of Diet and Physical Activity 2011–2013 (DANSDA 2011–2013) [67,68,69].
