# Supplementary material for: Changes in Diet and Physical Activity among 18–65-Year-Olds after the First National COVID-19 Lockdown in Denmark
Source: Nutrients. 2023 Mar 20;15(6):1480. doi: 10.3390/nu15061480 (PMC10054679; doi:10.3390/nu15061480)
Supplement: Supplementary file 1 [file nutrients-15-01480-s001.zip › Questionnaire.pdf]

Questionnaire, including the food frequency questionnaire (FFQ). Standard portion size of food and drinks are shown in parentheses

	1	2	3	4	5	6
**Sociodemographic**						
Sex	Male	Female				
Age *	18-34 y	35-49 y	50-65 y			
Education *	Basic school (<12 y)	Upper secondary school (12 y)	Vocational education (13 y, practical)	Short higher education (13-14 y)	Medium/long higher education Ph.D. (≥15 y)	
Region	Capital Region	Zealand	South Denmark	Central Jutland	North Jutland	
Household income*	<300,000 DKK	300,000-599,999 DKK	600,000-899,999 DKK	≥900000 DKK	Unknown/ Do not want to answer	
Family status *	Single without children <16 y	Single with at least one child <16 y	Couple without children <16 y	Couple with at least one child <16 y		

**Food frequency**					**Response options**						
**questionnaire**	**1**	**2**	**3**	**4**	**5**	**6**	**7**	**8**	**9**	**10**	**11**
The aim of the FFQ is to assess Danish adults' intake of food and drinks within the last 2 weeks. When filling out the questionnaire, it is important that you try to remember everything you have been eating and drinking in the last 2 weeks - also what you ate and drank for snacks etc.											
**Drinks:** For each type of drinks, frequency and portion size are recorded											
Frequency drinks (within the last 2 weeks)	Not within the last 2 weeks (0 days/14 days)	1 day within the last 2 weeks (1 day/14 days)	1 day a week for the last 2 weeks (2 days/14 days)	2-3 days a week for the last 2 weeks (5 days/14 days)	4-5 days a week for the last 2 weeks (9 days/14 days)	6-7 days a week for the last 2 weeks (13 days/14 days)	Unknown (0 days/14 days)				
Quantity drinks (per day)	Less than 1 glass/drink (0.5 glass/drink)	1 glass/drink (1 glass/drink)	2 glasses/ drinks (2 glasses/ drinks)	3 glasses/drinks (3 glasses/ drinks)	4 glasses/ drinks (4 glasses/ drinks)	5 glasses/ drinks (5 glasses/ drinks)	6 glasses/ drinks (6 glasses/ drinks)	7 glasses/ drinks (7 glasses/ drinks)	8 glasses/ drinks or more (8 glasses/ drinks)	Unknown (0 glasses/ drinks)	

Type of drink											
Milk	Milk, 0.3% fat or buttermilk (200 g)	Milk, 0.4% fat (200 g)	Milk, 1.5% fat (200 g)	Milk, 3.5% fat (200 g)	Chocolate milk (200 g)	Yoghurt drink (200 g)	Plant-based milk (200 g)				
Drinks	Juice (200 g)	Smoothie (200 g)	Cordial, sugar sweetened (200 g)	Cordial, sugar free (200 g)	Still water (200 g)	Sparkling water (200 g)	Iced tea or vitamin water (500 g)	Carbonated soft drinks, sugar sweetened (330 g)	Carbonated soft drinks, sugar free (330 g)	Protein drink (500 g)	Energy drink (250 g)
Alcohol	Alcohol free or light beer (330 g)	Beer (330 g)	Strong beer (330 g)	Red or white wine (150 g)	Liqueur wine (150 g)	Spirits (40 g)	Alcopops (330 g)				
Coffee	Espresso without milk/cream (50 g)	Espresso with milk/cream (60 g)	Black coffee without milk/cream (200 g)	Black coffee with milk/cream (200 g)	Café latte, cappuccino etc. (200 g)	Unknown/ Do not remember (200 g)					
Tea	Tea (black, green etc.) without milk/cream (200 g)	Tea (black, green etc.) (with milk/cream (200 g)	Herbal tea without milk/cream (200 g)	Herbal tea with milk/cream (200 g)	Unknown/ Do not remember (200 g)						
Added sugar in coffee and tea	Nothing (0 g)	<1 teaspoon (2.5 g)	1 teaspoon (5 g)	2 teaspoons (10 g)	3 teaspoons (15 g)	≥4 teaspoons (20 g)	Unknown (5 g)				

					Response options						
**Items**	**1**	**2**	**3**	**4**	**5**	**6**	**7**	**8**	**9**	**10**	**11**
**Food:** For each type of food or meal, frequency and portion size are recorded:											
Frequency food and meals (within the last two weeks)	0 times (0 times/ 14 days)	1-2 times (1.5 times/ 14 days)	3-4 times (3.5 times /14 days)	5-6 times (5.5 times/ 14 days)	7-8 times (7.5 times/ 14 days)	9-10 times (9.5 times/ 14 days)	11-12 times (11.5 times/ 14 days)	13-14 times (13.5 times/ 14 days)	≥ 15 times (15 times/ 14 days)		
Type of food											
Yoghurt	Yoghurt, 0.1-0.5% fat (200 g)	Yoghurt, 1.5% fat (200 g)	Yoghurt, 3.5% fat (200 g)	Yoghurt, 10% fat (150 g)	Soy yoghurt, vegan yoghurt etc. (200 g)	Cold buttermilk soup (200 g)					

Added fruit from manufacturer in yoghurt etc.	Never (0% of the time)	Rarely (25% of the time)	Regularly (50% of the time)	Often (75% of the time)	Always (100% the time)	Unknown				
Breakfast products	Oats (55 g)	Oatmeal/ Porridge (200 g)	Muesli (55 g)	*`Ymerdrys´* (25 g)	Cereals, cornflakes, Coco Pops etc. (30 g)					
Milk breakfast products	Milk, 0.1-0.5% fat (175 g)	Milk, 1.5% fat (175 g)	Milk, 3.5% fat (175 g)	Other (175 g)	Unknown (175 g)					
Bread	Rye bread (45 g)	White bread (45 g)	Whole grain bread (50 g)	Crisp bread (12 g)	Pita bread, flutes, flat bread etc. (65 g)	Biscuits (12 g)				
Slices of bread at a time	1 slice	2 slices	3 slices	4 slices	5 slices	6 slices	7 slices	≥ 8 slices		
Fat or butter on bread	Nothing (0 g)	Spreadable mixed product (5 g)	Butter (5 g)	Margarine (4 g)	Minarine (4 g)	Pesto (10 g)	Hummus (15 g)	Mayonnaise, Miracle Whip etc. (10 g)	Other (8 g)	Unknown (8 g)
Filling in closed or open-faced sandwiches (food groups)	Cheese	Cold cuts	Fish	Plant based cold cuts (20 g)	Egg (45 g)	Fruit and vegetables	Jam, honey, chocolate spread etc.			
Cheese on bread	Firm cheese 45+ (25 g)	Firm cheese 30+ (20 g)	Firm cheese 20+ (20 g)	Brie or camembert etc. (25 g)	Cottage cheese or cheese, unripened, smoked (35 g)	Cream cheese (25 g)	Vegan cheese (25 g)	Other (25 g)	Unknown (25 g)	
Cold cuts on bread	Liver paste, pate (20 g)	Ham, pork (saddle, smoked, boiled), roast beef (15 g)	Cold cuts of chicken/turkey (20 g)	Roast pork, pork (flank, spiced, cooked), sausage (salami), meatballs etc. (20 g)	Low fat cold cuts (15 g)	Other (20 g)	Unknown (20 g)			

Fish on bread	Smoked or marinated fish (40 g)	Fried fish (60 g)	Shrimp/mussels /crabs (40 g)	Tuna (50 g)	Mayonnaise salad e.g., tuna, mackerel, shrimp (40 g)	Cod, roe (40 g)	Other (35 g)	Unknown (35 g)			
Plant based cold cuts on bread (20 g)											
Egg on bread (45 g)											
Fruit and vegetables on bread	Potato, cucumber, tomato, avocado etc. (45 g)	Mayonnaise salad e.g., summer, Russian, curry (40 g)	Vegetable paste, pate, plant-based spread (25 g)	Fruit paste (40 g)	Other (30 g)	Unknown (30 g)					
Sweet spreads on bread	Jam (15 g)	Honey (10 g)	Chocolate spread, Nutella etc. (10 g)	Unknown (15 g)							

Photo series with portion sizes are shown for most of the meals

	Response options										
**Items**	**1**	**2**	**3**	**4**	**5**	**6**	**7**	**8**	**9**	**10**	**11**
Meals	Beef/veal	Pork	Lamb	Poultry (chicken/ turkey)	Fish or shellfish	Offal	Soup	Vegetarian meals			
Type of meal											
Potatoes (photo series no. 3)	Boiled potatoes (125 g)	Baked potatoes (150 g)	Fried potatoes (145 g)	French fries (145 g)	Scalloped potatoes, potato salad (180 g)	Mashed potatoes (200 g)					
Pasta/rice/beans (photo series no. 4)	Rice, bulgur, couscous (150 g)	Brown rice, whole grain bulgur or barley, spelt (150 g)	White pasta, noodles etc. (150 g)	Whole wheat pasta (150 g)	Dried beans, chickpeas, lentils (90 g)						

Pie/pizza/burger	Fried egg, scramble eggs, omelette etc. (100 g)	Pie with or without meat/fish (150 g)	Burger (240 g)	Pizza (200 g)	Pita, tortilla or similar (210 g)	Rice porridge (225 g)	Rice dish (risotto, paella) (220 g)		
Sauce	Melted butter (10 g)	Sauce with butter, cream or similar (75 g)	Low fat sauce without butter, cream or similar (75 g)	Cold sauce with sour cream (tzatziki) (30 g)	Ketchup, mustard or similar (15 g)	Pesto (15 g)	Oil/vinegar (15 g)	Thousand Island, Caesar dressing or similar (15 g)	Cheese (grated cheese/feta) (15 g)
Beef/veal (photo series no. 1)	Steak, roast beef or similar (100 g)	Beef patty (100 g)	Dishes with minced meat (meatballs) (100 g)	Stew with beef/veal (Bolognese, goulash, lasagne) (250 g)	Other dishes with beef/veal (150 g)	Unknown (100 g)			
Pork (photo series no. 1)	Roast pork (100 g)	Cutlet (100 g)	Pork filet/ham schnitzel (100 g)	Dishes with minced meat (100 g)	Stew with pork (250 g)	Other dishes with pork (150 g)	Unknown (100 g)		
Lamb (photo series no. 1)	Lamb chop/lamb cutlet (100 g)	Dishes with minced meat (100 g)	Stew with lamb (250 g)	Other dishes with lamb (150 g)	Unknown (100 g)				
Poultry (photo series no. 1)	Poultry (100 g)	Turkey (100 g)	Duck or goose (100 g)	Dishes with minced meat (100 g)	Stew with poultry (250 g)	Other dishes with poultry (150 g)	Unknown (100 g)		
Fish (photo series no. 3)	Lean fish (100 g)	Fatty fish (100 g)	Roe (75 g)	Shellfish (50 g)	Stew with fish and shellfish (250 g)	Sushi (250 g)	Other dishes with fish/ shellfish (100 g)	Unknown (100 g)	
Soup	Bouillon, chicken, vegetable (250 g)	Other soups with or without meat (250 g)	Unknown (250 g)						
Vegetarian	Plant based beef/pork (100 g)	Plant based chicken (100 g)	Plant based fish (100 g)	Vegetable or bean products (85 g)	Stew with vegetables (210 g)	Other vegetarian dishes (150 g)	Unknown (100 g)		

Meals	Mixed salads	Other vegetables not included in mixed salad (raw or cooked, e.g., cauliflower, carrots, cucumber, tomato etc., but not potatoes)		Fruit, including nuts (raw or prepared, e.g., apple, banana, berries, nuts etc.)							
Type of meal											
Mixed salad<br>(photo series no. 5)	With<br>meat/fish<br>(175 g)	With<br>rice/pasta/<br>bulgur<br>(175 g)	Only with<br>vegetables<br>and/or fruit<br>(175 g)	With both<br>meat/fish and<br>rice/pasta/<br>bulgur<br>(175 g)	With cheese<br>(175 g)	Other mixed<br>salad<br>(175 g)	Unknown<br>(175 g)				
Other vegetables<br>not included in<br>mixed salad<br>(photo series no. 6)	Mixed<br>vegetables<br>(100 g)	Cucumber<br>(50 g)	Tomato<br>(50 g)	Avocado<br>(45 g)	Corn or peas<br>(70 g)	Bell pepper<br>(50 g)	Green beans<br>(55 g)	Asparagus<br>(55 g)	Onion<br>(35 g)	Leek<br>(35 g)	Spinach<br>(70 g)
	Zucchini/<br>Eggplant<br>(55 g)	Mushroom<br>(40 g)	Carrot<br>(70 g)	Other root<br>vegetables<br>(60 g)	Cauliflower/<br>broccoli/<br>Brussel<br>sprouts<br>(80 g)	Cabbage<br>(75 g)	Butternut<br>squash/<br>Hokkaido/<br>sweet potato<br>(60 g)	Other<br>(60 g)	Unknown<br>(60 g)		
Fruit	Apple<br>(110 g)	Pear<br>(105 g)	Orange/<br>Clementine<br>(90 g)	Banana<br>(105 g)	Peach<br>(115 g)	Kiwi<br>(60 g)	Plum<br>(45 g)	Grapes<br>(80 g)	Berries<br>(75 g)	Dried fruit<br>(20 g)	Nuts<br>(25 g)
	Almonds<br>(25 g)	Pineapple<br>(35 g)	Melon<br>(100 g)	Other<br>(60 g)	Unknown<br>(60 g)						
Sweets and snacks<br>(photo series no. 7<br>and 8)	Chocolate<br>(30 g)<br>(photo series<br>no. 7)	Candy<br>(50 g)<br>(photo series<br>no. 7)	Whipped egg<br>white and<br>sugar with<br>chocolate<br>Covering<br>(20 g)	Ice cream<br>(75 g)	Popsicle and<br>sorbet<br>(50 g)	Cake<br>(70 g)<br>(photo series<br>no. 8)	Cake with<br>cream<br>(100 g)<br>(photo series<br>no. 8)	Fruit pie<br>(125 g)<br>(photo series<br>no. 8)	Danish<br>pastry<br>(75 g)	Sweet<br>biscuit<br>(30 g)	Pancakes<br>(75 g)
	Desserts<br>(100 g)	Potato crisps<br>(30 g)	Salted nuts<br>(35 g)	Popcorn<br>(40 g)	Cereal bar<br>(30 g)	Protein bar<br>(50 g)	Other<br>(100 g)				

**Physical activity**											
Moderate-to-vigorous intensity physical activity during the last 7 days	Moderate-to-vigorous intensity physical activity, (0-35 h/wk or >35 h/wk)	Moderate-to-vigorous intensity physical activity, (0, 15, 30 or 45 min/wk)	Vigorous intensity physical activity, (0-21 h/wk or >21 h/wk)	Vigorous intensity physical activity, (0, 15, 30 or 45 min/wk)							
TV and computer time during the last 7 days	Sedentary TV leisure, (0-16 h/day or >16 h/day)	Sedentary TV leisure, (0, 15, 30 or 45 min/day)	Sedentary computer leisure, (0-16 h/day or >16 h/day)	Sedentary computer leisure, (0, 15, 30 or 45 min/day)							
					**Response options**						
**Items**	**1**	**2**	**3**	**4**	**5**	**6**	**7**	**8**	**9**	**10**	**11**
**Stress level**											
Have you felt stressed during the last 2 weeks?	Not at all	Rarely	Occasionally	Often	All the time	Unknown/ Do not want to answer					
**Anthropometrics and weight change**											
Height without shoes (cm)											
Weight without clothes (kg)											
Weight change during the lockdown (March to May 2020)	Gained weight		Lost weight		Unchanged		Unknown				
Weight change (kg)	<1.0	1.0-1.9	2.0-2.9	3.0-3.9	4.0-4.9	≥5	Unknown/ Do not want to answer				
**Informed consent**	Would you give your consent to DTU to use your data for scientific publication? Yes or no										

* Composed variable

	Photo no. 1	Photo no. 2	Photo no. 3	Photo no. 4
Photo series no. 1	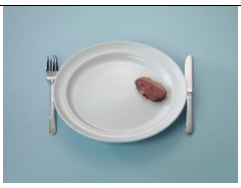<br>33% of standard portion size	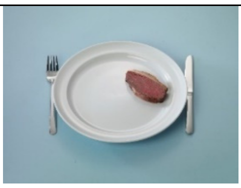<br>80% of standard portion size	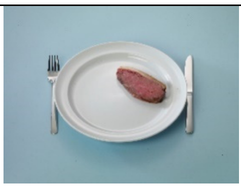<br>100% of standard portion size	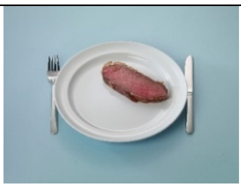<br>200% of standard portion size
Photo series no. 2	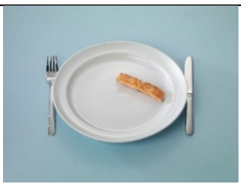<br>31% of standard portion size	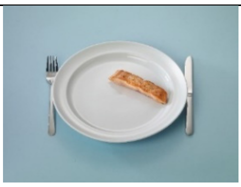<br>63% of standard portion size	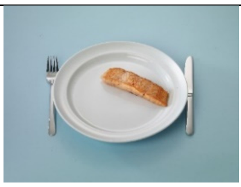<br>100% of standard portion size	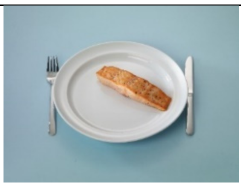<br>138% of standard portion size
Photo series no. 3	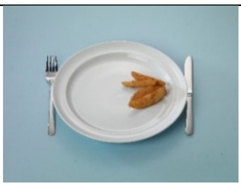<br>38% of standard portion size	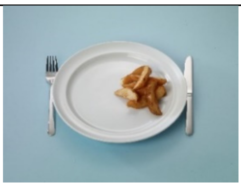<br>69% of standard portion size	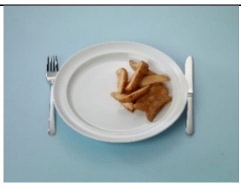<br>100% of standard portion size	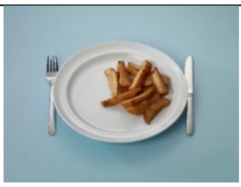<br>131% of standard portion size
Photo series no. 4	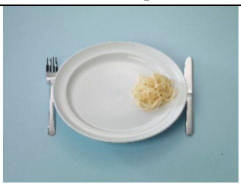<br>19% of standard portion size	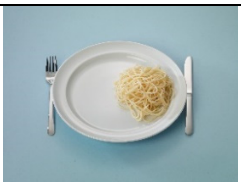<br>59% of standard portion size	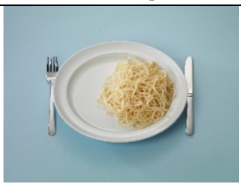<br>100% of standard portion size	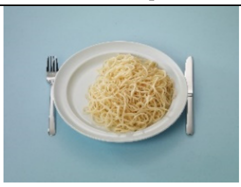<br>138% of standard portion size
Photo series no. 5	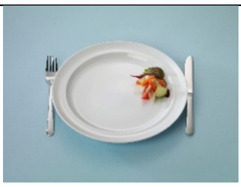<br>15% of standard portion size	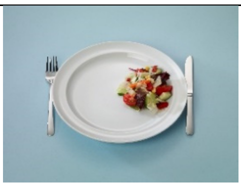<br>58% of standard portion size	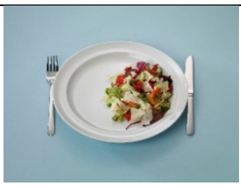<br>100% of standard portion size	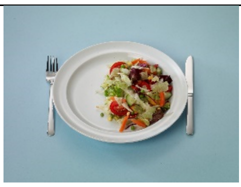<br>150% of standard portion size
Photo series no. 6	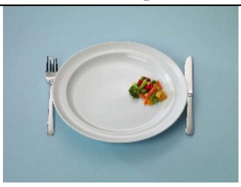<br>20% of standard portion size	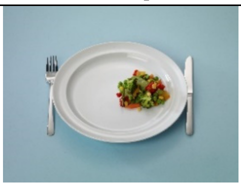<br>60% of standard portion size	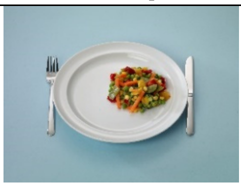<br>100% of standard portion size	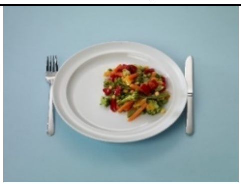<br>140% of standard portion size
Photo series no. 7	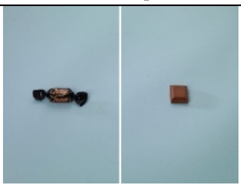<br>75% of standard portion size	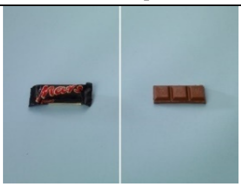<br>100% of standard portion size	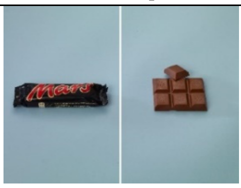<br>125% of standard portion size	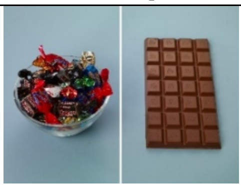<br>150% of standard portion size
Photo series no. 8	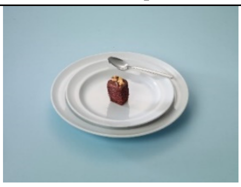<br>75% of standard portion size	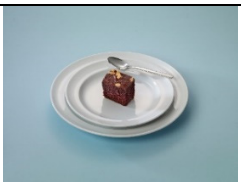<br>100% of standard portion size	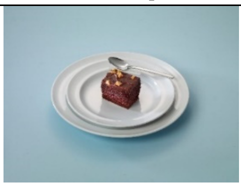<br>125% of standard portion size	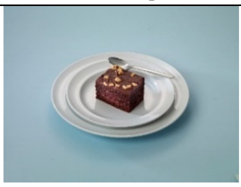<br>150% of standard portion size
