# Supplementary material for: Changes in Diet and Physical Activity among 18–65-Year-Olds after the First National COVID-19 Lockdown in Denmark
Source: Nutrients. 2023 Mar 20;15(6):1480. doi: 10.3390/nu15061480 (PMC10054679; doi:10.3390/nu15061480)
Supplement: Supplementary file 1 [file nutrients-15-01480-s001.zip › Supplementary Files.pdf]

Supplementary Materials

Outbreak management of COVID-19

During and 5-6 months after the first national lockdown in Denmark in 2020 (the first wave of the COVID-19 pandemic)

During the first national lockdown: March-April 2020

Measures for outbreak management of COVID-19 during the national lockdown in the period from 12 March to 13 April 13 2020 include temporary border control with entry ban, travel restrictions (advice against all non-essential international travel), closure of day care centers, schools, educational institutions and leisure activities, public sector employees are asked to work from home, encouragement for as many private employees as possible to work from home as well, ban on events etc. with more than 10 people, closure of night clubs, pubs, restaurants, cafés etc., closure of shopping centers etc., closure of sports and leisure venues etc., ban on liberal service professions (hairdressers, masseurs etc.) [67].

After the first national lockdown: September-October 2020

Measures for outbreak management of COVID-19 in the period after the lockdown from 19 September to 4 October 2020 include assembly ban of a maximum of 50 people, permitted opening hours for restaurants, bars and cafés limited to 22:00 in the evening, including 1 meter distance requirement for guests and use of face mask/visor (except when sitting down), grocery shopping is recommended to exclusively be carried out by one family member at a time, encouragement for public and private employees to work from home as much as possible, cancellation of social events in schools, leisure activities, educational institutions, workplaces, etc., 1 meter distance requirements and restriction of social interaction with others when participating in social activities and private gatherings (however, it is possible to conduct planned anniversaries such as weddings and confirmations for the closest circle of friends), but still maintaining an everyday life with work, education, restaurant visits, leisure activities etc. [68].

Table S1. Sociodemographic characteristics, weight status and diet among acceptable reporters and mis-reporters (under- and over-reporters) of energy during the lockdown.

	**Acceptable reporters** (n = 410)	**Mis-reporters** (n = 319)
Sex (% (n))		
Male	46.6 [a] (n = 191)	56.8 [b] (n = 187)
Female	53.4 [b] (n = 219)	43.2 [a] (n = 142)
Age (% (n))		
18-34 y	26.8 (n = 110)	28.9 (n = 95)
35-49 y	31.0 (n = 127)	33.4 (n = 110)
50-65 y	42.2 (n = 173)	37.7 (n = 124)
Education[#] (% (n))		
Short	40.5 (n = 166)	45.6 (n = 150)
Medium	13.2 (n = 54)	10.3 (n = 145)
Long	46.3 (n = 190)	44.1 (n = 145)
Weight status (% (n))	(n = 408)	(n = 326)
Underweight/normal weight	52.5 [b] (n = 214)	35.0 [a] (n = 114)
Overweight/obese	47.5 [a] (n = 194)	65.0 [b] (n = 212)
Diet (Mean ± SEM)	(n = 410)	(n = 329)
Dietary index score [&]	3.2 ± 0.0	3.3 ± 0.0
Total fat (E%)	34.7 ± 0.2	35.3 ± 0.3
Saturated fat (E%)	13.1 ± 0.1	13.0 ± 0.1
Carbohydrate (E%)	47.8 ± 0.3 [b]	46.1 ± 0.4 [a]
Added sugars (E%)	10.1 ± 0.3 [b]	8.8 ± 0.3 [a]
Dietary fiber (g/10 MJ)	23.5 ± 0.3	24.2 ± 0.4
Protein (E%)	15.5 ± 0.1 [a]	16.6 ± 0.2 [b]
Fruit and vegetables (g/10 MJ)	313 ± 8 [a]	347 ± 13 [b]
Whole grain (g/10 MJ)	71 ± 2	71 ± 2
Fish (g/10 MJ)	22 ± 1 [a]	26 ± 1 [b]
Red meat (g/10 MJ)	87 ± 2 [a]	102 ± 3[b]
Candy and snacks ″ (g/10 MJ)	113 ± 3	98 ± 3
Water (g/10 MJ)	971 ± 32 [a]	1361 ± 64 [b]
Sweetened drinks [¤] (g/10 MJ)	448 ± 30	548 ± 40
Alcoholic drinks [!] (g/10 MJ)	183 ± 16 [b]	152 ± 15 [a]

[a,b] Estimates with unlike superscript letters differed significantly ($p < 0.05$) between acceptable reporters and mis-reporters.

[#] Short (basic school, upper secondary school, vocational education), medium (short higher education) and long (medium/long higher education, Ph.D.).

[&] The overall diet quality was evaluated by means of a diet index score based on five food and nutrient guidelines from the Official Danish Dietary Guidelines 2013: Saturated fat (<10 E%), added sugars (<10 E%), fruit and vegetables (≥600 g/10 MJ/day), fish (≥350 g/10 MJ/week) and whole grain (≥75 g/10 MJ/day).

″ Sweets, chocolate, cake, biscuit, snack bar, ice cream, desserts and snacks.

[¤] Sugar sweetened and artificially sweetend soft drinks, energy drinks, cordials and iced tea.

[!] Beer, wine and other alcoholic drinks (liqueur, spirits, alcopops).

Table S2. Diet and physical activity among 18-65-year-olds in the Danish National Survey of Diet and Physical Activity 2011-2013 (DANSDA 2011-2013) and the Nordic Monitoring System 2014 (NORMO 2014).

	**DANSDA 2011-2013** § (Mean ± SEM)	**NORMO 2014** ^ (Mean ± SEM)
Diet	(*n* = 2560)	
Dietary index score #	3.3 ± 0.0	-
Energy (MJ/d)	9.8 ± 0.6	-
Total fat (E%)	37.8 ± 0.1	-
Saturated fat (E%)	15.0 ± 0.1	-
Carbohydrates (E%)	45.8 ± 0.1	-
Added sugars (E%)	8.7 ± 0.1	-
Dietary fiber (g/day)	22.0 ± 0.2	-
Protein (E%)	16.3 ± 0.1	-
Fruit and vegetables (g/day)	421 ± 4	-
Whole grain (g/day)	59 ± 1	-
Fish (g/day)	35 ± 1	-
Red meat (g/day)	137 ± 2	-
Candy and snacks ″ (g/day)	79 ± 1	-
Water (g/day)	968 ± 14	-
Sweetened drinks ¤ (g/day)	141 ± 5	-
Alcoholic drinks ! (g/day)	243 ± 7	-
Physical activity (PA)	(*n* = 2565)	(*n* = 1900)
Moderate-to-vigorous intensity PA (MVPA; h/wk)	4.0 ± 0.1	4.9 ± 0.1
Moderate intensity PA (MPA; h/wk)	2.6 ± 0.1	3.2 ± 0.1
Vigorous intensity PA (VPA; h/wk)	1.5 ± 0.0	1.7 ± 0.1
Physically inactive £ (% (n))	34.4	25.7
	(*n* = 2565)	(*n* = 1848)
Leisure screen time (h/day)	3.3 ± 0.0	4.1 ± 0.1
TV (h/day)	2.1 ± 0.0	2.4 ± 0.1
Computer (h/day)	1.2 ± 0.0	1.7 ± 0.0
Very high leisure screen time (>6 h/day; % (n))	8.8	16.4
	(*n* = 2536)	(*n* = 1755)
Sedentary leisure time $ (%)	3.7	3.6

§ Diet has been assessed with 7-d pre-coded food diaries [44] and physical activity with NPAQ [69].
^ The whole diet has not been assessed in NORMO 2014. Physical activity was assessed with NPAQ [41].
# The overall diet quality was evaluated by means of a diet index score based on five food and nutrient guidelines from the Official Danish Dietary Guidelines 2013: Saturated fat (<10 E%), added sugars (<10 E%), fruit and vegetables (≥600 g/10 MJ/day), fish (≥350 g/10 MJ/week) and whole grain (≥75 g/10 MJ/day).
″ Sweets, chocolate, cake, biscuit, snack bar, ice cream, desserts and snacks.
¤ Sugar sweetened and artificially sweetend soft drinks, energy drinks, cordials and iced tea.
! Beer, wine and other alcoholic drinks (liqueur, spirits, alcopops).
£ Failure to meet the PA guidelines.
$ Physically inactive with very high leisure screen time (>6 h/day).

Table S3. Seasonal variation between March-April and September for diet and physical activity among 18-65-year-old Danes in the Danish National Survey of Diet and Physical Activity 2011-2013 (DANSDA 2011-2013).

	**March-April** (Mean ± SEM)	**September** (Mean ± SEM)
Diet	(*n* = 387)	(*n* = 227)
Dietary index score [#]	3.3 ± 0.0	3.3 ± 0.1
Energy (MJ/d)	9.4 ± 1.5	10.1 ± 2.1
Total fat (E%)	37.9 ± 0.3	37.7 ± 0.4
Saturated fat (E%)	15.0 ± 0.2	15.1 ± 0.2
Carbohydrates (E%)	45.5 ± 0.4	46.4 ± 0.4
Added sugars (E%)	8.7 ± 0.3 [a]	9.1 ± 0.3 [b]
Dietary fiber (g/day)	21.4 ± 0.4	23.5 ± 0.6
Protein (E%)	16.6 ± 0.2 [b]	16.0 ± 0.2 [a]
Fruit and vegetables (g/day)	393 ± 11 [a]	441 ± 14 [b]
Whole grain (g/day)	59 ± 2	62 ± 3
Fish (g/day)	35 ± 2	33 ± 2
Red meat (g/day)	137 ± 4	143 ± 6
Candy and snacks [″] (g/day)	70 ± 3 [a]	85 ± 4 [b]
Water (g/day)	940 ± 34	971 ± 42
Sweetened drinks [¤] (g/day)	145 ± 13	133 ± 13
Alcoholic drinks [!] (g/day)	207 ± 16	256 ± 24
Physical activity	(*n* = 368)	(*n* = 274)
MVPA (h/wk)	4.2 ± 0.2	4.1 ± 0.3
MPA (h/wk)	2.6 ± 0.2	2.7 ± 0.2
VPA (h/wk)	1.6 ± 0.1	1.4 ± 0.1
Physically inactive [£] (% (n))	33.7	33.9
	(*n* = 375)	(*n* = 271)
Leisure screen time (h/day)	3.6 ± 0.1 [b]	3.1 ± 0.1 [a]
TV (h/day)	2.1 ± 0.1	2.0 ± 0.1
Computer (h/day)	1.4 ± 0.1 [b]	1.1 ± 0.1 [a]
Very high leisure screen time (> 6 h/day; %)	10.9	8.5
	(*n* = 367)	(*n* = 271)
Sedentary leisure time [$] (%)	4.4	3.0

[a,b] Estimates with unlike superscript letters differed significantly ($p < 0.05$) between groups in March-April and September. One-way ANCOVA with sex as covariate was used to analyze seasonal variation for diet due to a significant sex difference between the two groups. Independent sample *t*-test and chi square test were used to analyze seasonal variation for PA, as no sex and age differences were found between the two groups.

[#] The overall diet quality was evaluated by means of a diet index score based on five food and nutrient guidelines from the Official Danish Dietary Guidelines 2013: Saturated fat (<10 E%), added sugars (<10 E%), fruit and vegetables (≥600 g/10 MJ/day), fish (≥350 g/10 MJ/week) and whole grain (≥75 g/10 MJ/day).

[″] Sweets, chocolate, cake, biscuit, snack bar, ice cream, desserts and snacks.

[¤] Sugar sweetened and artificially sweetend soft drinks, energy drinks, cordials and iced tea.

[!] Beer, wine and other alcoholic drinks (liqueur, spirits, alcopops).

[£] Failure to meet the PA guidelines.

[$] Physically inactive with very high leisure screen time (>6 h/day).
